# Knowledge, Attitudes, and Practices of Parents Regarding Ophthalmological Screening of Preschool-Aged Children in Jazan, Saudi Arabia

**DOI:** 10.3390/clinpract14060198

**Published:** 2024-11-20

**Authors:** Mohamed Salih Mahfouz, Samy Shaban Mahmoud, Saleha Qaseem Haroobi, Latifah Mohammed Bahkali, Shahad Ibrahim Numan, Areen Mohsen Taheri, Ohoud Ali Hakami, Orjuwan Adel Zunquti, Sarah Mohammed Khered

**Affiliations:** 1 Family and Community Medicine Department, Faculty of Medicine, Jazan University, Jazan City, Jazan 45142, Saudi Arabia; 2 Public Health and Community Medicine Department, Faculty of Medicine, Al-Azhar University, Egypt; 3 Faculty of Medicine, Jazan University, Jazan City, Jazan 45142, Saudi Arabia

**Keywords:** children, eye screening, eye diseases, Saudi Arabia

## Abstract

Background: Children’s eye disorders are a major cause of irreversible vision loss. Delays in diagnosing eye problems in children are recurring problems that require quick attention. This study assesses parents’ knowledge, attitudes, and practices regarding the early ophthalmological screening of preschool-aged children in Jazan, KSA. Methods: An observational cross-sectional study was conducted among 522 parents of preschool-aged children in the Jazan region. A self-administered web-based questionnaire was randomly distributed to the parents via WhatsApp. The survey tool consisted of four main sections: socio-demographic data, knowledge about eye care, attitudes toward eye screening, and eye care practices. Results: Parents’ level of knowledge regarding children’s eye care was distributed as follows: low (21.5%), medium (40.2%), and high (38.4%). Parent gender, age, occupation, level of income, and nationality showed no statistically significant association with the knowledge level (*p* > 0.05 for all). However, parent education played a significant role (*p* = 0.013). Further, parents expressed a positive attitude toward the early screening of eye problems, as more than 90% agreed that early eye examinations for children reduce complications from visual problems and that the increased use of electronic devices requires early eye examinations. Almost 47.0% of the parents had examined their children’s eyes when they were between 1 and 5 years of age, compared with only 10.3% of parents of children less than 1 year of age. The multiple linear regression model for factors that predict knowledge level among the study participants showed that having a child undergo early screening is positively associated with an increased knowledge score (*p* < 0.05). Conclusions: Jazan parents showed a positive attitude toward the early screening of eye problems, and one-third had a high level of knowledge regarding children’s eye care. However, the proportion of those who practiced early eye screening was low. More health education is necessary to increase parents’ awareness regarding early eye care practices.

## 1. Introduction

Children’s eye disorders are a major cause of irreversible vision loss. The delayed diagnosis of eye problems in children is a recurring problem that requires quick attention. The World Health Organization (WHO) defines visual impairment as the degree to which a person is unable to discern fine details (visual acuity) using conventional techniques (such as the Snellen chart) [1]. Children’s vision impairment is a significant socioeconomic and public health issue worldwide [2].

Children under five years old, and some even older, are unable to effectively notice or explain ocular problems. Because of this, only parents can provide preventative care through early eye screening for their children. As a result, early identification and treatment of visual impairment at a young age can reduce the condition’s burden in adulthood [3].

Among children, congenital cataracts are a leading cause of vision impairment in developing countries. In middle-income countries, retinopathy of prematurity is more likely, while uncorrected refractive errors remain a leading cause of vision impairment globally [4,5].

The American Association of Pediatric Ophthalmology and Strabismus (AAPOS) advises annual screening tests for ages 3–5 [6], and the US Preventive Services Task Force (USPSTF) recommends at least one test between the ages of 3 and 5 [7]. Effective vision screening may increase issue detection and reduce referral costs [8]. Despite the importance of early eye screening, parental adherence to early child screening varies widely across countries, with rates ranging from 25% in the US to 83% in Germany among developed countries and even lower in developing countries [9,10].

Parents’ knowledge of potential visual disorders at a younger age is essential for seeking health counseling [11]. The literature suggests that Saudi parents’ knowledge of refractive errors, amblyopia, and eye care is limited [12,13,14,15]. Further, no research has been conducted on parents’ knowledge, attitudes, and practices regarding the ophthalmological screening of preschool-aged children in the Jazan region in southwest Saudi Arabia. As a result, this study will attempt to fill this gap by assessing the knowledge, attitudes, and practices of parents regarding ophthalmological screening for preschool children and, specifically, has the following objectives:

1. To assess parental knowledge of the importance of eye screening for preschool children (3–5 years) in the Jazan region (Saudi Arabia);
2. To determine parents’ attitudes toward having their children screened for eye diseases;
3. To determine whether parents have taken their children for eye screening and the reasons behind this;
4. To determine the socio-demographic factors that are relevant for parents to take their children to health centers for ophthalmological screening.

## 2. Methods

### 2.1. Study Design Setting and Population

An observational cross-sectional study was conducted among parents of preschool-aged children in the Jazan region in southwestern Saudi Arabia. The region is bordered to the north and east by the Asir region, to the west by the Red Sea, and to the south and southeast by the Republic of Yemen. According to the General Authority for Statistics, the total population in the region in 2010 was approximately 1.5 million, the total number of parents was 344,711, and the number of children between 0 and 9 years old was 236,943 (with 109,068 children with an age of 4 and under). The main inclusion criteria were parents who had children under the age of 5 and were living in the Jazan region. The exclusion criteria involved individuals who refused to participate or failed to complete the questionnaires, non-Jazan participants, or those who did not use the Arabic language. 

### 2.2. Sample Size and Design

Cluster random sampling was performed, covering sub-administrative regions within the Jazan region. We divided the Jazan region into sectors (north, south, east, and west). Five random clinics were selected from each sector to collect data. The study team met the study participants, and a survey link to the questionnaire was provided. The sample size was calculated using the sample size formula for a cross-sectional survey, *n* = [Z^2^P (1 − P)]/d^2^, where n is the initial sample size, and Z is the normal distribution for the 95% confidence level. Using the following parameters, *p* = estimated proportion =0.5, and d= desired precision = 4.5% for a 95% confidence level equal to 1.96, the initial sample size was 475. After accounting for a possible 10% non-response rate, the final sample size was 522 participants.

### 2.3. Method of Data Collection, Study Tools, and Piloting

Data were collected using a predesigned self-administered online questionnaire distributed to the parents using Google Forms. The questionnaire contained multiple parts: Part one included parents’ demographic data and six questions on gender, age, nationality, income, occupation, and educational level. Part two focused on parents’ knowledge of early eye care. Fifteen questions were used to measure this section, including the definition of lazy eye, strabismus, and refractive errors; knowledge about eye rubbing; and common eye problems. Part three covered parents’ attitudes toward children’s eye examinations. Ten questions were scored with agreement/disagreement/not sure and asked whether early eye screening reduces complications from vision problems in children: Does the increase in electronic devices used by children make early eye examinations necessary? Does wearing glasses improve your child’s vision? Part four of the questionnaire discussed the practices of parents in caring for children’s eyes. The questions included the following: Has your child had any type of eye exam? How often did the child undergo a routine eye examination? The pilot study was conducted among 30 people to test for suitability and the time needed to collect data after obtaining informed consent. The data were entered into SPSS and stored without any attempts to identify the subjects as the survey did not include personal information such as names, ID numbers, or specific details that could identify individuals. Based on this pilot study, Cronbach’s Alpha showed that the overall reliability was 0.906, knowledge was 741, attitude was 0.703, and practice was 0.676.

### 2.4. Operational Definitions and Study Variables

The parents’ level of knowledge regarding eye care was assessed using knowledge items in the questionnaire (10 questions). Each correct response was given a score of 1, while incorrect responses were scored as 0. A total knowledge score was computed by summing individual responses for each participant. High knowledge was defined as a total score equal to or above 75. A score of less than 50 indicated a low level of knowledge. Independent variables involved the following: (1) socio-demographic factors, including age, education, and occupation, the number of children, and family monthly income; and (2) child-related factors such as child age, gender, and weight. The dependent variables were the total knowledge score, parents’ attitudes toward children’s eye examinations, and parents’ practices concerning children’s eye examinations.

### 2.5. Data Management and Statistical Analysis

After the data collection stage was completed, the data were downloaded from Google Forms, manually verified, and coded within an Excel sheet. The data were transformed and analyzed using the Statistical Package for the Social Sciences (SPSS) version 29. Data were analyzed using descriptive and inferential statistics. The Chi-Squared test assessed associations, and multiple linear regression identified the most important predictors of patient knowledge, using the parent knowledge score as the dependent variable. A *p*-value less than 0.05 indicated statistical significance.

### 2.6. Ethical Consideration

This study was conducted according to the ethical standards of the Saudi Bioethics Committee. All respondents were asked to read/understand and sign the study’s informed consent form before joining this study. All the data collected were used only for research purposes and kept confidential. Ethical clearance for this was obtained from the University of Jazan Ethical Committee (reference no.: REC-44/06/469, (approval date: 4 January 2022).

## 3. Results

The estimated response rate for this survey was 96.4% (503 out of 522). The participants’ socio-demographic characteristics are presented in Table 1. Most participants in the study are mothers, accounting for 65.6% of the sample. Almost 97.6% of the participants are Saudis, and 51.7% live in rural areas. The age distribution indicates that most participants (43.1%) are between 25 and 39 years old. Workers in the government sector make up 47.5% of the participants, while 64.2% have a university qualification. Table 1 shows that the parents’ level of knowledge regarding children’s eye care was distributed as follows: low 20.5%, medium 40.2%, and high 38.4%. Parent gender, age, occupation, level of income, nationality, and whether the child had any eye problems showed no statistically significant association with the level of knowledge (*p* > 0.05 for all). However, parent education played a significant role (*p* = 0.013): 39% of parents with university-level education had the highest knowledge score, whereas only 17.2% of parents with primary school-level education had a high level of knowledge.

Parents who knew that neglecting visual problems leads to permanent blindness in children constituted around 56.9% of the study participants, without significant differences between fathers (56.6%) and mothers (57%) (*p* = 0.674). Further, 80.9% of fathers and 87.9% of mothers knew that early lazy eye treatment leads to better results (*p* = 0.109). The percentage of parents who were keen to pay attention to their children when rubbing their eyes was 73.4%, with 76.3% of fathers compared to 71.8% of mothers, but without a statistically significant difference (*p* = 0.280). Almost 81.1% of parents identified that redness in the eye and resulting tears required visiting the ophthalmologist. In comparison, 88.7% responded yes to taking the child to an ophthalmologist if there was difficulty in distinguishing distant/near shapes. Most parents (85.7%) could identify refractive errors, and 79.3% knew about strabismus, but only 27.2% knew about lazy eyes. There were no significant differences between the responses of the fathers and mothers across all statements and definitions in Table 2 (*p* > 0.05 for all).

Table 3 presents the parents’ attitudes toward early screening for eye problems. The parents expressed a positive attitude toward the early screening of eye problems, as more than 90% agreed with the statements that early eye examinations for children reduce the complications of visual problems and that children’s increased use of electronic devices requires an early eye examination. Almost half (52.1%) agreed that wearing glasses can improve their child’s vision, while 41.4% were not sure. Furthermore, 68.6% agreed with the statement that academic performance is affected by visual problems. Once again, parents expressed a positive attitude, and 86.9% agreed that early eye examination can prevent or treat permanent damage from lazy eyes/blurriness and blurred vision. Less than half (48.5%) agreed that children (under the age of six) are more likely to have strabismus, and 45.9% were not sure. There were no significant differences between the responses of the fathers and mothers across all statements in the table (*p* > 0.05 for all).

Table 4 illustrates the parents’ practices regarding early eye screening for their children. We found that almost half of fathers (49.7%) took their children to ophthalmology clinics for examinations, which was significantly higher than mothers (37.0%) (*p* = 0.006). Further, almost 46.7% of the parents examined their children between 1 and 5 years of age compared to only 10.3% for children who were less than one year of age. Regarding the question of how often their child had undergone a routine eye examination, responses were as follows: every year, 16.1%; more than once a year, 20.7%; every two years, 8%; every five years, 2.3%; and not sure, 21.1%. The parents’ reasons for conducting the screening were as follows: the child plays with toys from a close distance, 61.4%; the child repeatedly rubs his eyes, 46.2%; the child’s head is tilted to one side frequently, 31.2%; the child suffers from a squint in the eye, 26.7%; the child complains of double vision (one thing is perceived as two things), 24.2%; and the child suffers from frequent tears and red eyes, 45.7%. There were no significant differences between the responses of the fathers and mothers across all statements in the table (*p* > 0.05 for all).

**Figure 1:**
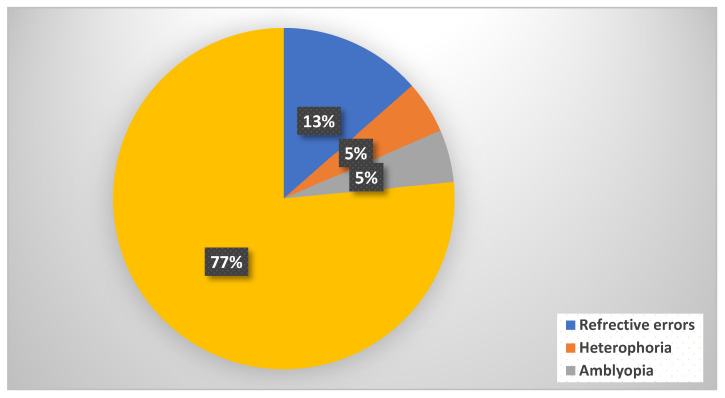
Common eye problems among the children in this study (*n* = 503).

Figure 1 illustrates the common eye problems reported by the parents among their children. Refractive errors were the most prevalent ailment among children in Jazan (13%), besides the majority (77%) of research participants, who claimed that their children did not suffer from any eye problems.

**Figure 2:**
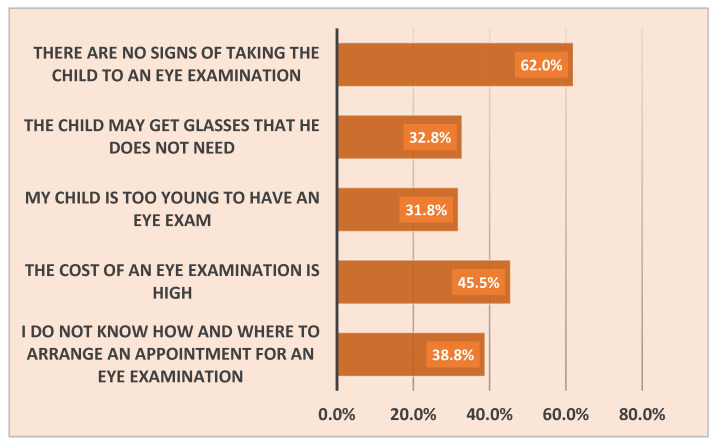
Parents’ reasons for not taking their child to an eye examination (*n* = 503).

Figure 2, on the other hand, shows the parents’ reasons for not taking their children for an eye examination. Overall, half of the fathers (50.3%) and more than half of the mothers (63.0%) did not take their children for an eye examination. The most common reason was that there were no signs requiring an eye screening (62%), followed by the high cost of eye examinations (45.5%). Other causes are summarized in Figure 2.

Table 5 provides the multiple linear regression model for factors that predict knowledge scores among the study participants. Having a child undergo early screening is positively associated with an increased knowledge score, while having a child with any eye problem is negatively associated with a high score (*p* < 0.05 for both). Being a female is associated with an increase in knowledge scores compared to being a male, but the association lacks statistical significance (*p* > 0.05).

## 4. Discussion

To the best of our knowledge, this study is the first to assess the knowledge, attitudes, and practices of parents regarding early ophthalmological screening and testing for preschool-aged children in Jazan. Understanding these factors in Saudi Arabia is crucial for promoting the early detection and treatment of eye conditions.

Our study found that the level of knowledge about children’s eye care was distributed as follows: low (20.5%), medium (40.2%), and high (38.4%). We also compared our results with earlier research. A study in Arar city showed that 56.7% of parents had adequate knowledge [14]. However, a study in Al-Medina revealed that 78.2% of parents had poor knowledge [15]. Another study conducted among 1070 participants in Riyadh found that 91.9% of parents had low knowledge of childhood eye disorders [16]. These differences may be due to variations in the knowledge evaluation methods, sample differences, and populations.

Our results suggest that parents with university-level education had the highest knowledge scores, whereas only 17.2% of parents who had a primary school-level education received a high score. A recent study conducted in 2020 to determine parents’ knowledge and practices regarding child eye health care in Saudi Arabia showed that university graduates and parents under 20 years of age had better knowledge of eye care [17]. By contrast, another cross-sectional study conducted in Swaziland in 2018 showed no significant association between the level of education and knowledge of child eye health care [18]. Most parents with a high level of knowledge also had high incomes. This outcome makes sense, as wealthier families have better access to health care and are better health-educated. Parents between the ages of 25 and 39 who have postgraduate degrees, work in the private sector, and reside in cities tend to possess better knowledge. Lastly, parents with only a primary education degree possess the lowest level of knowledge. This finding can be explained by the hypothesis that adults with university education have better knowledge owing to their education, leading them to read, watch, or listen to health education content on social media or participate in community awareness events hosted in shopping centers and cities. Among the unexpected results was a higher percentage of knowledge possessed by parents with an intermediate level of education. This may be because parents with an average level of education tend to compensate for their deficiency by increasing their knowledge in various fields, including issues which concern their children’s health.

Our results indicate that the attitudes of parents toward wearing glasses to improve vision were as follows: 52.1% agreed, 6.6% disagreed, and 41.4% were not sure. In comparison, another study conducted in Saudi Arabia investigated whether wearing glasses if a child needs them under the age of seven will make eyes and vision stronger; the findings showed that 47.5% agreed, 16% disagreed, and 36.5% were not sure [19]. Parents’ positive attitudes toward the use of glasses is an essential step for children’s eye care [20].

This study showed that 46.7% of parents had examined their children between the ages of 1 and 5, while only 10.3% had performed so before 1 year of age. These findings may be the reason that eye issues can be seen as more significant as children grow older and begin to exhibit more overt visual habits, like reading or playing. Infants frequently do not show outward symptoms of visual problems, delaying examinations. Further, parents frequently concentrate on important aspects of a baby’s health throughout infancy, such as vaccines, growth, and developmental milestones. In the absence of obvious risk factors or a family history, routine eye exams might not be stressed as an integral element of early treatment.

The linear regression documented that an association between having a child undergo early screening is positively associated with an increased knowledge level. Much research has emphasized the importance of parents’ awareness in avoiding children’s visual problems [11,18,21,22]. Parents with lower levels of education have said that they do not see the need for their children to undergo an eye examination. By contrast, parents with higher levels of education have reported the opposite. This implies that individual educational attainment may substantially impact a parent’s understanding of their children’s ocular well-being. In addition, those with lower levels of education have more financial limitations and more limited access to eye care services than those with higher academic credentials.

Although the parents in this study showed a positive attitude toward the early screening of eye problems, only one-third had a high level of knowledge regarding children’s eye care. The proportion of those who practice early eye screening was low. One important tactic to raise public awareness of standards for eye care is health promotion. Using the preferred informational channels of parents—campaigns and billboards—might be one method to accomplish this. The present study’s consequences should open the eyes of ophthalmologists, public health officials, and policymakers. Health education materials that target the parents of children with no eye problems are required as a prevention strategy. On the other hand, addressing highly common disorders like myopia, associated amblyopia, and strabismus requires screening and integrating primary eye care for early identification and prompt management when necessary.

This study has some limitations that should be considered. Firstly, as this was a cross-sectional study, it was challenging to accurately determine the relationship between parents’ practices regarding ophthalmologic screening and specific study outcomes, such as whether the child had undergone any type of visual examination or when the child had had their first examination. Additionally, this study relied on a self-administered online questionnaire. Therefore, the fact that all of the responses came from parents who are proficient users of social media and the internet suggests that awareness may be overrepresented. Furthermore, the study was restricted to the Jazan region, a small area with similar knowledge and attitudes among residents. Lastly, the sample size was relatively small in relation to Jazan’s population size. Despite these limitations, this research is the first attempt to establish knowledge, attitudes, and practices regarding early ophthalmological screenings in the Jazan region. Moreover, this study also targeted a particular preschool age range to assess community awareness of early eye screening and its potential to prevent several persistent disease-related problems, such as lazy eye.

## 5. Conclusions

Jazan parents showed a positive attitude toward the early screening of eye problems, and one-third had a high level of knowledge regarding children’s eye care. However, the proportion of those who practiced early eye screening was low. More health education is necessary to increase parents’ awareness regarding early eye care practices.

## Figures and Tables

**Table 1:** Parents’ background characteristics and level of knowledge regarding children’s eye care according to selected characteristics (*n* = 503).

Characteristics		All Parents		Level of Knowledge						*p*-Value
				Low		Moderate		High		
		N	%	N	%	N	%	N	%	
Gender	Male	173	(34.4)	37	(21.4)	73	(42.2)	63	(36.4)	0.765
	Female	330	(65.6)	71	(21.5)	129	(39.1)	130	(39.4)	
Age Groups	18–24 years	78	(15.5)	19	(24.4)	29	(37.2)	30	(38.5)	0.547
	25–39 years	217	(43.1)	45	(20.7)	80	(36.9)	92	(42.4)	
	40–54 years	186	(37.0)	38	(20.4)	85	(45.7)	63	(33.9)	
	55 years or more	22	(4.4)	6	(27.3)	8	(36.4)	8	(36.4)	
Educational Level	Postgraduate	33	(6.6)	6	(18.2)	12	(36.4)	15	(45.5)	0.013
	University	323	(64.2)	60	(18.6)	137	(42.4)	126	(39.0)	
	High school	89	(17.7)	19	(21.3)	37	(41.6)	33	(37.1)	
	Intermediate	19	(3.8)	6	(31.6)	4	(21.1)	9	(47.4)	
	Primary	39	(7.8)	15	(51.7)	9	(31.0)	5	(17.2)	
Occupation	Governmental sector	241	(47.9)	52	(21.6)	104	(43.2)	85	(35.3)	0.617
	Private sector	46	(9.1)	10	(21.7)	14	(30.4)	22	(47.8)	
	Retired	129	(25.6)	29	(22.5)	52	(40.3)	48	(37.2)	
	Not working	87	(17.3)	17	(19.5)	32	(36.8)	38	(43.7)	
Monthly Income	Less than 5000	131	(26.0)	31	(23.7)	49	(37.4)	51	(38.9)	0.790
	5000 to 10,000	146	(29.00	42	(28.8)	54	(37.0)	50	(34.2)	
	10,000–15,000	123	(24.5)	20	(16.3)	57	(46.3)	46	(37.4)	
	15,000 and more	103	(20.5)	15	(14.6)	42	(40.8)	46	(44.7)	
Nationality	Saudi	491	(97.6)	104	(21.2)	200	(40.7)	187	(38.1)	0.231
	Non-Saudi	12	(2.4)	4	(33.3)	2	(16.7)	6	(50.0)	
Does your child have an eye problem	Yes	112	(22.3)	28	(25.0)	50	(44.6)	34	(30.4)	0.138
	No	391	(77.7)	80	(20.5)	152	(38.9)	159	(40.7)	
Parents’ Overall Level of Knowledge				108	(20.5)	202	(40.2)	193	(38.4)	

**Table 2:** Parents’ knowledge about certain items of eye care and early screening (*n* = 503).

Items		All Parents		Gender				
				Fathers		Mothers		*p*-Value
		N	%	N	%	N	%	
Can neglecting visual problems lead to permanent blindness in children?	Yes	286	(56.9)	98	(56.6)	188	(57.0)	0.674
	No	26	(5.2)	11	(6.4)	15	(4.5)	
	I do not know	191	(38.0)	64	(37.0)	127	(38.5)	
Does early treatment of lazy eyes lead to better results?	Yes	430	(85.5)	140	(80.9)	290	(87.9)	0.109
	No	9	(1.8)	4	(2.3)	5	(1.5)	
	I do not know	64	(12.7)	29	(16.8)	35	(10.6)	
Constant rubbing of the eye requires visiting an ophthalmologist.	Yes	369	(73.4)	132	(76.3)	237	(71.8)	0.280
	No	134	(26.6)	41	(23.7)	93	(28.2)	
Eye redness and tears require visiting an ophthalmologist.	Yes	408	(81.1)	145	(83.8)	263	(79.7)	0.262
	No	95	(18.9)	28	(16.2)	67	(20.3)	
If the child’s eyes do not move in unison in the same direction, a visit to the ophthalmologist is required.	Yes	433	(86.1)	153	(88.4)	280	(84.8)	0.269
	No	70	(13.9)	20	(11.6)	50	(15.2)	
Difficulty distinguishing distant/near shapes.	Yes	446	(88.7)	153	(88.4)	293	(88.8)	0.907
	No	57	(11.3)	20	(11.6)	37	(11.2)	
Definition of lazy eye.	Incorrect	366	(72.8)	129	(74.6)	237	(71.8)	0.511
	Correct	137	(27.2)	44	(25.4)	93	(28.2)	
Definition of strabismus.	Incorrect	104	(20.7)	41	(23.7)	63	(19.1)	0.225
	Correct	399	(79.3)	132	(76.3)	267	(80.9)	
Definition of refractive errors.	Incorrect	72	(14.3)	29	(16.8)	43	(13.0)	0.256
	Correct	431	(85.7)	144	(83.2)	287	(87.0)	

**Table 3:** Parents’ attitudes toward early screening of eye problems (*n* = 503).

Statements		All Parents		Gender				
				Fathers		Mothers		*p*-Value
		N	%	N	%	N	%	
Early eye examinations for children reduce the complications of visual problems.	Agree	461	(91.7)	161	(93.1)	300	(90.9)	0.327
	Not sure	33	(6.6)	11	(6.4)	22	(6.7)	
	Disagree	9	(1.8)	1	(0.6)	8	(2.4)	
The increased use of electronic devices by children requires the need for an early eye examination.	Agree	456	(90.7)	158	(91.3)	298	(90.3)	0.781
	Not sure	42	(8.3)	14	(8.1)	28	(8.5)	
	Disagree	5	(1.0)	1	(0.6)	4	(1.2)	
Will wearing glasses improve your child’s vision?	Agree	262	(52.1)	79	(45.7)	183	(55.5)	0.107
	Not sure	208	(41.4)	82	(47.4)	126	(38.2)	
	Disagree	33	(6.6)	12	(6.9)	21	(6.4)	
Academic performance is affected by visual problems.	Agree	345	(68.6)	115	(66.5)	230	(69.7)	0.738
	Not sure	107	(21.3)	40	(23.1)	67	(20.3)	
	Disagree	51	(10.1)	18	(10.4)	33	(10.0)	
Can early eye examinations of your child prevent or treat permanent damage from lazy eyes/blurriness and blurred vision?	Agree	437	(86.9)	150	(86.7)	287	(87.0)	0.614
	Not sure	60	(11.9)	22	(12.7)	38	(11.5)	
	Disagree	6	(1.2)	1	(0.6)	5	(1.5)	
Do you think that children (under the age of six) are more likely to have strabismus?	Agree	244	(48.5)	83	(48.0)	161	(48.8)	0.854
	Not sure	231	(45.9)	79	(45.7)	152	(46.1)	
	Disagree	28	(5.6)	11	(6.4)	17	(5.2)	
Do you think that early detection of myopia and farsightedness in children contributes to reducing complications?	Agree	438	(87.1)	150	(86.7)	288	(87.3)	0.741
	Not sure	60	(11.9)	22	(12.7)	38	(11.5)	
	Disagree	5	(1.0)	1	(0.6)	4	(1.2)	
Does your child sitting close to the TV make you take him for an early eye examination?	Agree	397	(78.9)	136	(78.6)	261	(79.1)	0.511
	Not sure	89	(17.7)	29	(16.8)	60	(18.2)	
	Disagree	17	(3.4)	8	(4.6)	9	(2.7)	
Does a genetic vision problem in the family increase the importance of early examination?	Agree	427	(84.9)	145	(83.8)	282	(85.5)	0.887
	Not sure	65	(12.9)	24	(13.9)	41	(12.4)	
	Disagree	11	(2.2)	4	(2.3)	7	(2.1)	
Does having an eye examination center nearby increase your demand for an early examination of your child?	Agree	434	(86.3)	150	(86.7)	284	(86.1)	0.155
	Not sure	59	(11.7)	17	(9.8)	42	(12.7)	
	Disagree	10	(2.0)	6	(3.5)	4	(1.2)	

**Table 4:** Parents’ practices regarding the early eye screening of their children (*n* = 503).

Factors		All Parents		Gender				
				Fathers		Mothers		*p*-Value
		N	%	N	%	N	%	
Has your child ever had any type of visual examination?	Yes	208	(41.4)	86	(49.7)	122	(37.0)	0.006
	No	295	(58.6)	87	(50.3)	208	(63.0)	
Age of child when first eye exam was performed?	Less than one year	25	(10.3)	8	(8.5)	17	(11.5)	0.758
	One year to less than five	113	(46.7)	45	(47.9)	68	(45.9)	
	Five years and more	104	(43.0)	41	(43.6)	63	(42.6)	
How many times has the child undergone a routine eye examination?	Every year	42	(16.1)	19	(19.0)	23	(14.3)	0.196
	More than once a year	54	(20.7)	20	(20.0)	34	(21.1)	
	Every two years	21	(8.0)	9	(9.0)	12	(7.5)	
	Every five years	6	(2.3)	5	(5.0)	1	(0.6)	
	Only when the child reports a problem	83	(31.8)	29	(29.0)	54	(33.5)	
	Not sure	55	(21.1)	18	(18.0)	37	(23.0)	
Reasons for conducting eye screening.	The child plays with toys from a close distance	154	(61.4)	61	(62.9)	93	(60.4)	0.692
	The child rubs his eye frequently	115	(46.2)	42	(46.2)	73	(46.2)	0.994
	The child’s head tilts to one side frequently	77	(31.2)	28	(30.8)	49	(31.4)	0.916
	The child suffers from a squint in the eyes	66	(26.7)	29	(31.5)	37	(23.9)	0.189
	The child complains of double vision (one thing perceived as two)	61	(24.2)	23	(24.2)	38	(24.2)	0.998
	The child suffers from frequent tears and has red eyes	117	(45.7)	44	(45.8)	73	(45.6)	0.974

**Table 5:** Multiple linear regression model for factors associated with the total scores related to parents’ knowledge as a dependent variable (*n* = 503).

Variable	Coef	SE Coef	*t*-Value	*p*-Value
Constant	70.72	2.57	27.48	0.000
Gender				
Male (ref)	0.98	1.91	0.51	0.609
Child with any eye problem				
No (ref)	−6.07	2.45	−2.48	0.014
Health center				
No (ref)	2.30	2.03	1.13	0.257
Conducted early screening				
No (ref)	5.45	2.10	2.60	0.010
Residence				
Urban (ref)	−0.18	1.87	−0.09	0.925

Abbreviations: Coef = regression coefficient; SE Coef = standard error of the coefficient; and ref = reference category.

## Data Availability

This study has no additional supporting data to share.
